# Design of an intelligent wearable device for real-time cattle health monitoring 

**DOI:** 10.3389/frobt.2024.1441960

**Published:** 2024-11-21

**Authors:** Zhenhua Yu, Yalou Han, Lukas Cha, Shihong Chen, Zeyu Wang, Yang Zhang

**Affiliations:** ^1^ Department of Mechanical Engineering, Imperial College London, London, United Kingdom; ^2^ Dyson School of Design Engineering, Imperial College London, London, United Kingdom; ^3^ Department of Mechanical Engineering at the Technical University of Munich, Munich, Germany; ^4^ Department of Surgery and Cancer, Imperial College London, London, United Kingdom; ^5^ Department of Electrical and Electronic Engineering, Imperial College London, London, United Kingdom

**Keywords:** cattle health monitoring, non-invasive temperature sensing, behavioral classification, precision livestock farming, machine learning

## Abstract

In the realm of precision cattle health monitoring, this paper introduces the development and evaluation of a novel wearable continuous health monitoring device designed for cattle. The device integrates a sustainable solar-powered module, real-time signal acquisition and processing, and a storage module within an animal ergonomically designed curved casing for non-invasive cattle health monitoring. The curvature of the casing is tailored to better fit the contours of the cattle’s neck, significantly enhancing signal accuracy, particularly in temperature signal acquisition. The core module is equipped with precision temperature sensors and inertial measurement units, utilizing the Arduino MKR ZERO board for data acquisition and processing. Field tests conducted on a cohort of ten cattle not only validated the accuracy of temperature sensing but also demonstrated the potential of machine learning, particularly the Support Vector Machine algorithm, for precise behavior classification and step counting, with an average accuracy of 97.27%. This study innovatively combines real-time temperature recognition, behavior classification, and step counting organically within a self-powered device. The results underscore the feasibility of this technology in enhancing cattle welfare and farm management efficiency, providing clear direction for future research to further enhance these devices for large-scale applications.

## 1 Introduction

Smart wearable devices have revolutionized cattle health management. They provide continuous, real-time monitoring of health indicators like body temperature and movement patterns (Decandia et al. 2017; Lees et al. 2019; Hoffmann et al., 2013). This technology enables early detection of illnesses, improves the efficiency of livestock management, and enhances animal welfare. By providing detailed health data, these devices aid in making informed decisions, thus reducing the risk of disease outbreaks and increasing overall farm productivity.

Body temperature is an important factor for health monitoring, and this is because cattle are known for their remarkable ability to maintain a constant body temperature, and the accurate assessment of this temperature is of paramount importance in understanding their health and wellbeing (Hodnik et al., 2021). However, cattle commonly suffer from three prevalent underlying diseases, each with distinct impacts on their wellbeing and temperature. Firstly, bovine mastitis adversely affects milk production and quality, resulting in significant economic losses (Kim et al., 2019; Dutta et al., 2022). Infected cattle exhibit an average body temperature increase of 2.64°C compared to healthy ones. Secondly, the bovine respiratory disease complex (BRDc) is a costly issue, primarily caused by bacterial infections (Cusack et al., 2003). Elevated body temperature, exceeding 40°C, is a critical symptom, but early diagnosis is challenging. Heat stress, the third concern, arises due to excessive heat exposure, affecting milk production and fertility (Bagath et al., 2019; Idris et al., 2021). Measuring rectal temperature above 39°C and respiration rates over 60 beats per minute are indicators of heat stress. Temperature assessment remains crucial for disease diagnosis and animal wellbeing.

Traditionally, thermistors, semiconductor devices sensitive to temperature, are used for core temperature measurement (Hoffmann et al., 2013; Church et al., 2014; Kou et al., 2017). However, they require lookup tables for accurate readings and are non-linear. Rectal thermometers are common but necessitate cattle restraint, inducing stress-induced hyperthermia and reducing accuracy. Contact sensors, while less invasive, involve rectal, ear canal, or vaginal placement, each with limitations. Swallowed sensors face digestive system constraints and environmental influences. Infrared (IR) technology offers a non-contact alternative, and Infrared thermography (IRT) captures surface temperature by detecting emitted infrared radiation, which has been used since 2005 for early detection of laminitis, leg injuries, and stress responses in cattle (Wang et al., 2021; Stewart et al., 2017). It is also effective in non-invasively detecting udder temperature variations in mastitis cases. The technology’s advantage lies in its continuous monitoring capability, providing a comprehensive temperature profile over time.

Behavior detection technology also plays a pivotal role in the health monitoring of cattle, offering valuable insights into their wellbeing (Rahman et al., 2018; Riaboff et al., 2019; Benaissa et al., 2019b). These devices, often equipped with accelerometers and gyroscopes, are designed to meticulously track cattle movements. Accelerometers have emerged as essential tools for comprehensively monitoring cattle behavior. These devices, prized for their small size, affordability, and the ability to record high-resolution data for extended periods, play a pivotal role in understanding individual and social behaviors among cattle. By incorporating accelerometers and gyroscopes, researchers can meticulously track cattle movements, allowing for the precise identification and classification of behaviors like grazing, resting, and distress signals (Benaissa et al., 2019a). Machine learning algorithms, such as SVM Wang et al. (2018), K-Nearest Neighbors (KNN) Tian et al. (2021), and Multilayer Perceptrons (MLP) Balasso et al. (2021), further refine the analysis of behavioral patterns, offering valuable insights into cattle wellbeing and social dynamics.

Accelerometer technology has demonstrated its effectiveness across a range of applications (Shamoun-Baranes et al., 2012; Martiskainen et al., 2009). Pioneering studies on free-ranging wild animals, including vultures and Eurasian badgers, have utilized tri-axial accelerometer data. Domesticated animals, such as goats and dairy cows, have also been subjects of research employing accelerometers to classify behaviors. However, despite the promising potential of accelerometers, some challenges remain. These include issues related to the accurate recognition of specific behaviors, computational costs associated with certain machine learning algorithms like SVM, and practical considerations such as battery life and data retrieval methods for bio-telemetry sensors (Fuentes et al., 2020).

The significance of device sustainability and uninterrupted operation in cattle behavior monitoring is paramount (Cox, 2002; Rivero and Daim, 2017). It is imperative to note that prior research often inadequately considered this critical aspect, with earlier models lacking a systematic approach to ensuring continuous functionality. In contrast, contemporary devices prioritize sustainability, frequently incorporating rechargeable batteries and, in some innovative designs, integrating solar panels to extend battery life. These advancements not only reduce the ecological footprint of monitoring technology but also mitigate logistical challenges associated with frequent battery replacements Alonso et al., 2020). The past oversight underscores the urgency of current efforts to address sustainability, as these modern device innovations reshape cattle behavior monitoring, promising continuous and effective operation, and offering profound insights into animal wellbeing and livestock management.

To effectively address the identified shortcomings, this paper introduces a novel wearable continuous health monitoring device designed for cattle health assessment. The primary contribution of this research is the innovative integration of real-time temperature recognition, behavior classification, and step counting within a self-powered device. This device combines a sustainable solar-powered module, real-time signal acquisition and processing, and a storage module within an animal ergonomically designed curved casing for non-invasive cattle health monitoring. Furthermore, our distinctive design innovation lies in the fact that the animal ergonomically designed curved casing is tailored to better fit the contours of the cattle’s neck, significantly enhancing signal accuracy, particularly in temperature signal acquisition by comparing with the rectal temperature. Moreover, we have achieved precise step counting for cattle health assessment. Field tests conducted on a cohort of ten cattle not only validated the accuracy of temperature sensing but also demonstrated the potential of machine learning, particularly the Support Vector Machine (SVM) algorithm, for precise behavior classification and step counting, with an average accuracy of 97.27%.

The structure of this paper is organized as follows: Section 1 provides a summary of the latest research on temperature detection and behavior monitoring using wearable smart devices for cattle health and highlights the existing research gap. Section 2 details the device’s design and methodology, highlighting its modular construction and integration of sensors. Section 3 presents the experimental setup and results from the deployment on cattle, focusing on the performance of the machine learning algorithms, particularly SVM, for behavior analysis. Section 3 also discusses the findings, challenges, and future enhancements of the device. The paper concludes with Section 5, summarizing the key contributions and potential advancements in livestock health monitoring.

## 2 Device design and methods

**FIGURE 1:**
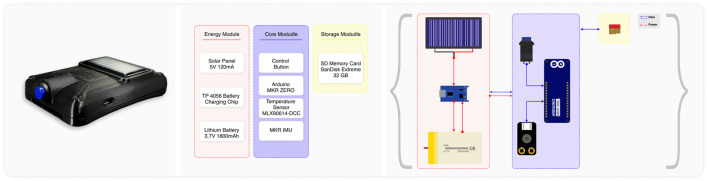
Schematic of the intelligent health monitoring device with interchangeable components. The device consists of three main modules: the Energy Module, Core Module, and Storage Module. Interconnections between modules are indicated, highlighting the power and data transfer paths.

**FIGURE 2:**
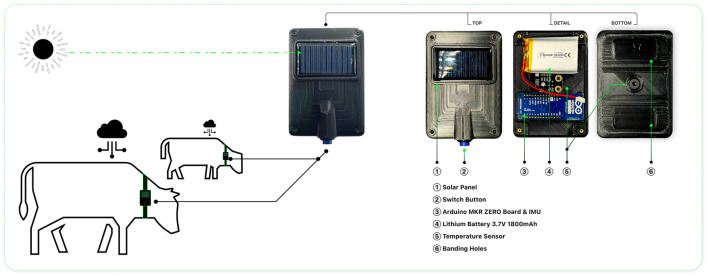
Intelligent wearable health monitoring system with integrated solar-powered sensor device.

The overall framework diagram for intelligent health monitoring in cattle is depicted in Figure 1. It primarily consists of three components: the energy supply module, the system control and sensing module, and the storage module. The detailed construction of the developed intelligent device is illustrated in Figure 2. In this section, we elaborate on the integration of the modular electronic device depicted in Figure 1 and its application in an agricultural setting as illustrated in Figure 2.

### 2.1 Integrated sensor integration

The Core Module lies at the heart of the device’s application in cattle health monitoring, anchored by the Arduino MKR ZERO board which orchestrates the data processing. The board’s MLX90614-DCC sensor is a centerpiece for non-invasive health monitoring, offering a measurement accuracy of ± 0.5°C within the range of −70°C to +380°C. This precision is crucial for detecting febrile conditions in livestock, which can signify infection or illness. The sensor’s high emissivity coefficient makes it adept at reading temperatures from various animal coats and skin types, and its fast response time of less than 500 milliseconds allows for near-instantaneous data acquisition.

Complementing the temperature sensor, the MKR ZERO board is equipped with a state-of-the-art IMU, capable of detecting movements in six degrees of freedom. This sensor’s performance metrics include an angular rate of ± 245/ ± 500/ ± 2000° per second (dps) on the gyroscope axis and an acceleration range of ± 2/ ± 4/ ± 8/ ± 16 g on the accelerometer axis. Such detailed motion tracking is instrumental for gait analysis in livestock, which can aid in the early detection of lameness or behavioral changes associated with distress or environmental changes.

### 2.2 Sustainable power supply subsystem design

The continuous operation of these high-precision sensors is assured by the Energy Module, which provides a consistent energy supply via its solar panel and battery system. The device’s Energy Module showcases a meticulously engineered power system, comprising a solar panel with a 5 V 120 mA output, which ensures optimal energy harnessing from sunlight. This energy is judiciously stored in a robust lithium battery with an impressive 1800 mA h capacity, ready to sustain the device’s operations through variable light conditions. The TP4056 chip within this module safeguards the battery against the rigors of overcharging and deep discharging, thus preserving the longevity of the 1800 mA h battery which sustains the device’s operations.Based on multiple real-world tests, the device can run for approximately 120 h on an 1800 mA h battery. Considering that the solar panel can provide some additional power, the actual operating time could be even longer. This will provide green and sustainable power to the monitoring system.

Data accrued from these sensors is logged by the Storage Module onto an SD memory card, designed to handle the extensive data generated over long periods. The card’s storage capacity ensures that no critical information is lost, enabling comprehensive analysis of temperature fluctuations and movement patterns over time.

This sophisticated sensor integration, backed by robust performance indicators, ensures that the device not only monitors the real-time state of livestock health, but also builds a historical database to inform long-term animal welfare strategies. The precision and reliability of these sensors are vital in translating complex biological and environmental data into actionable insights for farm management.

### 2.3 Enhanced utility through curved design

As shown in Figure 1, the device’s innovative design is encapsulated within a shell of precise dimensions, measuring exactly 10 × 7 cm. This compact and curved housing is engineered with a dual purpose: to minimize its physical footprint for ease of attachment to livestock without causing discomfort and to serve functional needs critical to farm operation. The curvature is more than aesthetic; it is a deliberate choice that ensures the device conforms to the natural lines of an animal’s body, promoting stability and accuracy in sensor readings. This design consideration is vital in environments like farms, where dust and debris are prevalent, as the curved surface inherently reduces the accumulation of such particulates.

**FIGURE 3:**
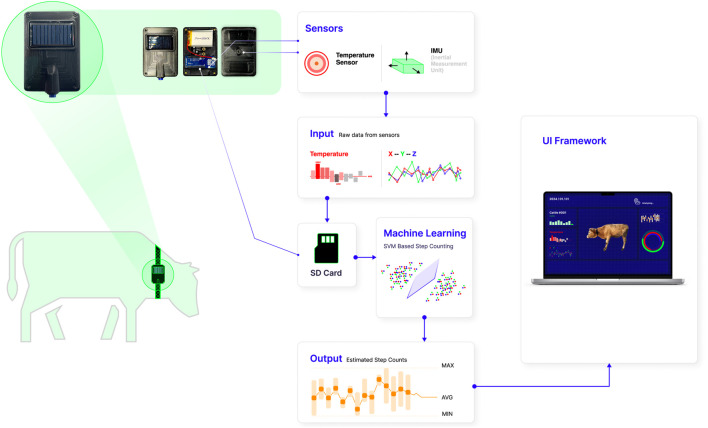
The experimental cattle equipped with smart wearable devices for temperature measurements. Temperature and acceleration information was collected in real-time.

Furthermore, the arc-shaped exterior plays a strategic role in enhancing the device’s durability and functionality. As shown in Figures 2, 3, the curvature of the shell allows it to fit more snugly against the curved flanks of livestock, thus ensuring better stability and sensor readings It acts as a shield, deflecting impacts from farm animals or machinery, thereby maintaining the device’s structural integrity and operational reliability. The curvature also contributes to the thermal management of the device. It facilitates a more uniform heat distribution and accelerated heat dissipation, safeguarding sensitive internal components like the MLX90614-DCC temperature sensor from the effects of ambient temperature fluctuations.

In summary, the seamless integration of the energy, core, and storage modules within a curved, ergonomically designed shell demonstrates a harmonious balance between form and function. This design synergy renders the device exceptionally suited to meet the demands of modern agriculture. It stands as a testament to thoughtful engineering, aimed at delivering reliable performance and valuable data insights while enduring the rigors of the farming environment.

## 3 Experiments and results

The smart wearable devices were affixed to 10 cattle, numbered from $C_{1}$ to $C_{10}$ , and conducted experiments for a minimum of 3 hours for data collection. All animal experiments were approved by the local livestock regulatory authorities, and the animal experiments were conducted in Weifang City, Shandong Province, China.Simultaneously, we measured the rectal temperature of the cattle to assess the accuracy of our temperature measurements. While rectal temperature measurement can affect the behaviour of cattle, its effect on the temperature measurements of the smart wearable device is negligible. This is because, as depicted in Figure 6, the device was affixed to the lateral side of the cattle’s neck using a slightly elastic nylon cord.

The reason for selecting the lateral side of the neck of the cattle was due to the relatively sparse hair in this region, increased blood circulation in the neck, and ease of attachment. The entire temperature measurement environment was situated inside the livestock barn on the farm, aimed at minimizing the impact of direct sunlight on the animals’ skin temperature and mitigating the potential interference of direct sunlight on the infrared sensors of the wearable device. In order to facilitate the measurement of rectal temperature in cattle and to affix and retrieve experimental devices, all the test cattle were tethered within a designated area using ropes. Despite the limited range of movement, the acceleration data still allowed for precise identification of the cattle’s activity behaviors.

### 3.1 Body temperature monitoring

Ensuring the health of cattle is vital in modern agriculture. Accurate body temperature measurement is key. By integrating our advanced technology into livestock management, we empower farmers to monitor and ensure the wellbeing and productivity of their cattle based on our wearable device, contributing to a thriving agricultural industry.

#### 3.1.1 Data processing: Smoothing method

Infrared Thermography (IRT) data can be highly variable and extremely sensitive to environmental conditions, as highlighted in previous research. Consequently, IRT data may exhibit significant noise, contain erroneous readings, and yield inaccurate results if a robust analysis methodology is not implemented. To accommodate the use of the MLX90614 sensor, a 1 s rolling median filter was employed on the raw temperature readings from consecutive frames. This smoothing technique was chosen to minimize the influence of outliers within the dataset. The 1 s window duration was selected because it effectively smooths the data over a sufficient period, reducing the impact of brief events that might disrupt accurate temperature measurements from the eye’s ocular region. These brief events may include animal blinks, head shaking, or other distortions such as camera refocusing. Importantly, this approach is adaptable to different hardware configurations, as the smoothing window adjusts based on the camera’s frame rate.

**FIGURE 4:**
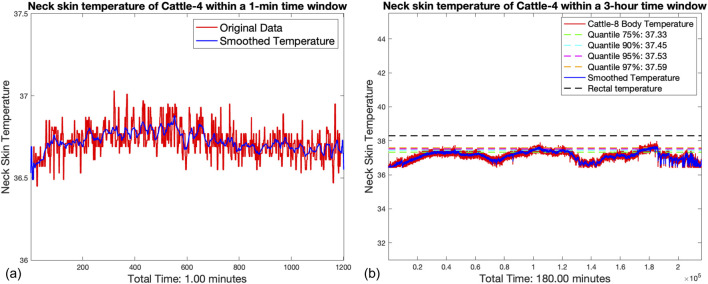
**(A)** Instantaneous neck skin temperature readings of Cattle-4 within a brief 1 min interval. **(B)** 3 h continuous neck skin temperature monitoring of the Cattle-4, comparing with the rectal temperature baseline.

Figure 4A illustrates the instantaneous neck skin temperature readings of Cattle-4 within a brief 1 min interval. The graph reveals the inherent variability of the raw IRT data (shown in red), which fluctuates rapidly between 36.5°C and 37.5°C. After applying a 1 s rolling median smoothing technique, the resultant data (indicated in blue) exhibit a more stable pattern, with smoothed values predominantly ranging between 36.7°C and 37.2°C. This technique successfully mitigates transient environmental effects and measurement artifacts without significantly altering the underlying temperature trends.

In Figure 4B, we extend our analysis to a broader 3 h time frame for Cattle-4. The raw data, without any filtering, display pronounced peaks and troughs, highlighting the sensitivity of IRT to short-term disturbances such as head movement or camera refocusing. Post-processing with the rolling median filter reveals a smoother temperature curve that closely shadows the median quantile lines at 37.33°C (75%), 37.45°C (90%), 37.53°C (95%), and 37.59°C (97%). The smoothed temperatures settle marginally below the rectal temperature baseline which is 38.3°C, which is a common comparative standard in livestock health monitoring. Taking into consideration that the epidermal temperature of homeothermic animals typically runs slightly lower than their rectal body temperature, the obtained detection results are reasonable. With the application of suitable correction algorithms, we can achieve long-term accurate monitoring of cattle health.

#### 3.1.2 Evaluation of skin vs rectal temperature variability in cattle

**FIGURE 5:**
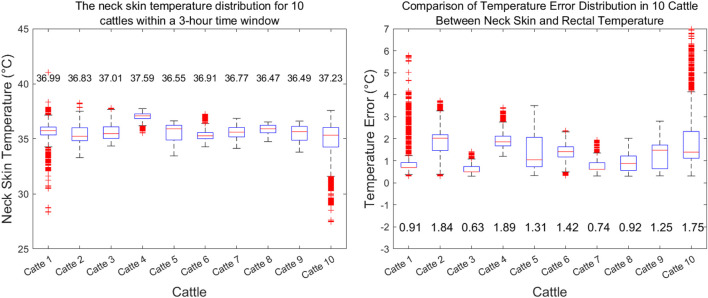
**(A)** Boxplot depicting the distribution of neck skin temperatures for a sample of 10 cattle over a 3 h observation period. Each box represents the interquartile range (IQR) of temperatures for an individual animal. **(B)** Boxplot comparison of the temperature measurement error between neck skin and rectal temperatures for the same cohort of 10 cattle. The boxes illustrate the distribution of the temperature differences for each animal, with the median error depicted by the central line in each box.

Transitioning to a population-level perspective, Figure 5A presents a boxplot of neck skin temperature distributions for a cohort of 10 cattle. Across this sample, median temperatures lie within a narrow band from 36.5°C to 37.2°C, aligning closely with the expected normal range. Notable are the individual variations, with Cattle $C_{1}$ showing a lower median of approximately 36.3°C, and Cattle $C_{10}$ displaying a higher median near 37.3°C. The interquartile ranges and the spread of outliers provide insight into the individual differences within the herd, potentially attributable to a myriad of factors including age, coat thickness, and activity levels.

Figure 5B addresses the critical question of how neck skin temperature correlates with the rectal body temperature, traditionally measured rectally. The boxplots denote the discrepancy between the two measurement sites for each of the 10 cattle. The median errors range from −0.2°C to 1.8°C, suggesting that while IRT provides a reliable non-invasive temperature assessment, certain individual and environmental factors can introduce a degree of error. Notably, Cattle-1 $C_{1}$ and Cattle-10 $C_{10}$ exhibit the largest deviations, which could be indicative of measurement anomalies or genuine physiological differences.

The robustness of the rolling median as a smoothing method for IRT data is reinforced by the data presented. By consistently reducing the noise and minimizing the influence of outliers, the method ensures that the temperature readings are representative of the animal’s true thermal state. It is crucial to acknowledge the inherent limitations of IRT, especially its sensitivity to environmental conditions. However, our methodological approach, by averaging over a 1 s window, demonstrates an effective balance between data fidelity and practicality for real-world monitoring.

The processed IRT data, as demonstrated in Figures Figures 4, 5, offer valuable insights into the thermal patterns of cattle, essential for health and welfare monitoring. The choice of a 1 s rolling median smoothing method has proven to be particularly suited to this application, allowing for the capture of physiologically relevant temperature changes while discounting transient environmental or sensor-related noise.

### 3.2 Cattle behaviour monitoring and classification

#### 3.2.1 Data collection

Data collection took place at a commercial cattle farm in China, where the cattle were housed in a cubicle shed. The cattle were fed with dry hay. A total of 10 cattle that showed no signs of severe lameness or other potential diseases affecting their behavior were selected for this research. Cattle were chosen and equipped with collars in the afternoon. Video recordings were employed at the same time to document the cattle’s activities, facilitating later data labeling for behavioral classification. Expanding on the analysis of outliers, it is important to consider potential factors contributing to temperature anomalies in cattle. Low temperatures, as observed in Cattle-1 with a median of approximately 36.3°C, may be indicative of issues such as head shaking or movement, which can cause the sensor to momentarily lose proper contact with the skin. Conversely, short-term elevated temperatures, as seen in Cattle-10 with a median near 37.3°C, could result from direct exposure to sunlight or other environmental factors.

Cow behavioral activities were annotated by observers (ZY) based on video recordings, with each cow wearing a sensor collar. IMU (Inertial Measurement Unit) data for each behavioral activity were manually labeled according to the following criteria.

1. *Feeding:* Cattle eating grass in a designated area;
2. *Grazing:* Cattle lowering its head to eat the hay;
3. *Walking:* Cattle moving from one location to another;
4. *Lying resting:* Cattle lying down in a restful state;
5. *Standing resting:* Cattle standing still and resting;
6. *From Stand up to lying down(transition):* Cattle transitioning from standing to lying down.
7. *From lying down to stand up (transition)* Cattle moving from a lying position to standing up.

It is important to note that certain less frequent or short-duration activities, such as drinking and scratching behaviors in livestock, are not explicitly identified in this categorization. However, it is crucial to emphasize that these rare activities and events may still hold significant biological relevance in assessing health and welfare conditions. Hence, while not attempting to classify these in the present context, future research should consider developing methodologies to detect such infrequent behaviors, acknowledging their potential importance in comprehensive animal welfare studies.

In this paper, we selectively focus on activities of interest to validate the activity classification algorithm. Data pertaining to the movements of ten cattle, labeled 1 to 10, were collected for a duration of 3 hours per animal. Notably, cattle numbered 1, 2, and three were subject to continuous temperature data collection over a 24 h period. This concurrent temperature monitoring serves a dual purpose: it provides an opportunity to verify the state of temperature surveillance while also facilitating the validation of the motion classification algorithm.

Upon completion of data collection, we proceeded to calibrate the IMU data based on video recordings that commenced simultaneously. A 20 s window was employed for data calibration. This process resulted in the calibration of 9 hours of motion data for cattle numbered 8, 9, and 10, which was subsequently utilized for training the machine learning algorithm.

**FIGURE 6:**
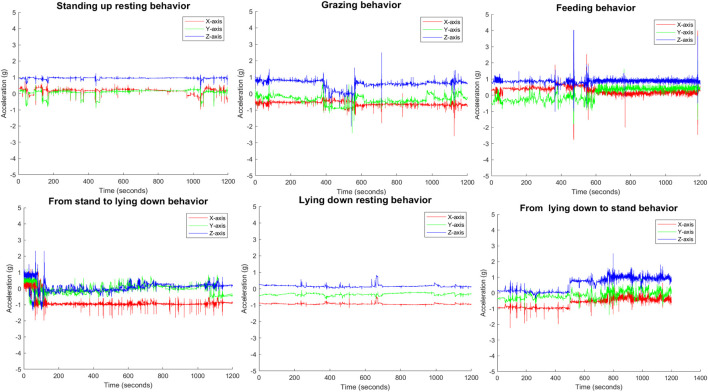
Various Motion States of Cattle based on the IMU data: Illustrating Standing Rest, Grazing, Feeding, Transition from Standing to Lying Down, and Lying Down Resting.

Figure 6 presents a time series example of the raw triaxial accelerometer output during various behaviors of a single cattle, including lying and standing resting, grazing, and feeding, as well as during transitions from lying to standing postures. The accelerometer outputs for lying and standing rest positions are qualitatively similar, reflecting minimal overall movement in both behaviors. Notably, during feeding, there is a distinct pattern of changes in the three-axis acceleration, corresponding to the cattle’s head movements up and down and back and forth. While Figure 6 is a representative example, similar qualitative patterns of accelerometer outputs were also observed in other cattle within the study. These qualitative observations provide a useful intuitive starting point for identifying the most appropriate features to be included in the classification algorithm.

#### 3.2.2 Feature characteristics extraction

Raw data collected from 10 cattle were used for the data pre-processing, which included acceleration, angular velocity, and magnetometer data acquired by the IMU on the device. An important aspect of classifying multidimensional time series is the extraction of a low-dimensional representation of the original input space. Therefore, we selected Mean (F1), Variance (F2), Root Mean Square (F3), Skewness (F4), Kurtosis (F5), Energy (F6), and Integral of absolute value (F8) as features. We use the manually labeled data set to train the machine learning classifier. The 10 s windows of cattle data are represented as a 9 × 8 matrix, indicating the seven features for each time series. Features are repeatedly extracted from the time series obtained from the 10 cattle in the experiment, forming a complete feature space for supervised behavior classification.

#### 3.2.3 Cattle behavior classification and identification

In this research, we conducted an experiment to classify cattle activities, employing several classification algorithms including K-Nearest Neighbors (KNN), Naive Bayes, Multilayer Perceptrons (MLP), multi-class Support Vector Machine (SVM), random forest (RF) Shi et al. (2024) and Binary Classification Trees (BCT). The experiment utilized data from specifically tagged cattle, identified by the numbers $C_{8}$ , $C_{9}$ , and $C_{10}$ . This data, already labeled, provided a solid foundation for testing and evaluating the efficacy of the various classification methods in accurately identifying different activities of the cattle. The diverse range of algorithms was chosen to explore and compare their respective strengths and weaknesses in the context of animal behavior analysis.

To assess the efficacy of classification algorithms, we evaluated two key metrics: the sensitivity and precision of the classification outcomes. In the realm of standard statistical process control, sensitivity (denoted as $C_{\text{Sen}}$ ) and precision (referred to as $C_{\text{Pre}}$ ) are delineated as follows:

$$C_{\text{Sen}}=\frac{\text{TP}}{\text{TP}+\text{FN}},\;C_{\text{Pre}}=\frac{\text{TP}}{\text{TP}+\text{FP}}.$$ (1)

in which, TP (True Positive) refers to the count of instances where the behavior of interest was accurately classified by the algorithm, as confirmed by visual observation. FN (False Negative) denotes the occurrences where the behavior of interest, although observed in reality, was incorrectly categorized as a different behavior by the algorithm. FP (False Positive) represents the instances where the algorithm inaccurately classified a behavior as being of interest, despite it not being observed in reality.

We comprehensively discuss the effectiveness of data collected by monitoring devices in accurately identifying cattle behavior, thereby verifying the reliability of these devices across various behavioral categories including feeding, resting, walking, grazing, and standing resting. As shown in Table 1, an in-depth performance assessment of five machine learning algorithms reveals the crucial importance of data quality in ensuring algorithmic accuracy. Notably, the SVM algorithm excelled in both sensitivity and precision assessments, achieving an average sensitivity of 93.83% and precision of 89.88% , underscoring the key role of high-quality data input in generating reliable classification results. Furthermore, the diversity and comprehensiveness of the data provide a robust training foundation for machine learning algorithms, ensuring the effectiveness and robustness of monitoring devices in different scenarios.

**TABLE 1:** Performance comparison of Six Machine-Learning Algorithms on Identical Cattle Data.

Behaviour class	Performance	KNN	Naive bayes	MLP	SVM	BCT	RF
Feeding	Sensitivity	88.08%	83.06%	75.06%	92.53%	80.06%	89.47%
	Precision	81.53%	88.26%	79.34%	90.12%	85.45%	87.59%
Lying resting	Sensitivity	79.05%	85.16%	68.29%	93.85%	82.47%	90.28%
	Precision	84.75%	80.26%	76.98%	88.54%	79.69%	85.82%
Walking	Sensitivity	86.13%	81.95%	70.86%	95.73%	78.33%	92.06%
	Precision	82.67%	79.02%	77.19%	91.27%	82.11%	88.74%
Grazing	Sensitivity	87.91%	80.36%	73.47%	94.38%	83.55%	91.15%
	Precision	85.42%	87.98%	75.31%	89.17%	81.23%	86.92%
Standing resting	Sensitivity	78.64%	84.23%	69.87%	92.64%	81.29%	88.94%
	Precision	83.52%	78.67%	74.58%	90.43%	80.11%	86.68%
Average	Sensitivity	84.00%	82.95%	71.51%	93.83%	81.14%	90.38%
	Precision	83.58%	81.04%	76.68%	89.88%	82.12%	87.15%

The comprehensive analysis indicates that the monitoring devices are not only capable of accurately recording everyday cattle behaviors, but the data they provide is vital for training machine learning algorithms, as evidenced by the high performance of these algorithms. Hence, it can be concluded that monitoring devices are highly reliable in collecting cattle behavior data, significantly enhancing the precision of livestock management. These findings are crucial for guiding the development of future agricultural monitoring technologies and lay a solid foundation for developing more efficient behavioral monitoring solutions. Although each algorithm has its unique strengths, for example, the RF algorithm also obtained the second-best performance, SVM provides the best balance in most scenarios, emphasizing the importance of considering data characteristics and application contexts when selecting appropriate machine learning algorithms.

### 3.3 Cattle step accounting

#### 3.3.1 SVM-based step counting in cattle

In cattle health management, it is vital to monitor locomotor patterns, as a decrease in step frequency, especially reduced step count, often signals health issues. Conditions like lameness, commonly caused by hoof or joint complications, are indicated by decreased mobility. Metabolic disorders such as ketosis or acidosis, along with reproductive challenges or estrus, are also linked to changes in activity levels. Reduced movement in cattle, similar to human responses to illness, generally reflects health deterioration. Therefore, careful monitoring of cattle’s movements is essential for the early detection and management of various health conditions. By analyzing variations in step counts over time, farmers can identify early signs of ailments like lameness or metabolic diseases, whose symptoms vary with disease progression, and increased activity that might signal estrus. This ongoing observation helps differentiate normal behavioral changes from health-related issues, enabling timely intervention and treatment.

Therefore, based on the manually annotated data for cattle and utilizing the same temporal signal feature extraction methodology as employed in subsection.3.2.2 features, we employed a binary SVM approach for the step counting test in cattle. During this process, we meticulously optimized key parameters of the SVM model, including the selection of the kernel type, adjustment of the regularization parameter C, and other parameters applicable to specific kernels (such as Gamma for the RBF kernel). Through this approach, we ensured a good fit of the model to the training data while preventing overfitting, thereby guaranteeing the generalizability of the model.

**FIGURE 7:**
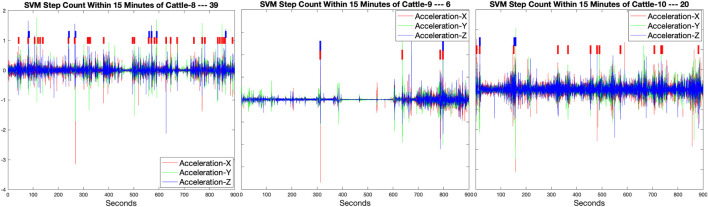
Cattle numbered $C_{8}$ , $C_{9}$ , and $C_{10}$ were manually marked and their step counts were recorded in three sample windows, each with a duration of 15 min. The identified step counts for each cattle are 39, 6, and 20, respectively. Each box plot in the figure represents a single step taken by the cattle at that moment. The blue box plots are particularly useful for illustrating instances when the cattle engaged in consecutive walking behavior.

Figure 7 presents the statistics of the step-counting results for these three cattle numbered $C_{8}$ , $C_{9}$ , and $C_{10}$ using the optimized SVM model, thereby demonstrating our reliance on the SVM model for accurately counting gait steps. From the graph, it can be observed that, for the 15 min time windows of Cattle numbered $C_{8}$ , $C_{9}$ , and $C_{10}$ , the identified step counts for each cattle are 39, 6, and 20, respectively. Within this time window, the actual manually counted steps were 45, 6, and 24 for the respective cattle. Comparative analysis reveals that the algorithm demonstrates relatively accurate gait recognition and counting for cattle within short time windows, achieving an average accuracy of approximately 86.67% . Additionally, the algorithm is capable of recognizing and counting consecutive gaits, as evidenced by the red and blue box plots in the figure. Notably, when cattle exhibit lower activity levels, the algorithm achieves higher accuracy due to the prominence of temporal features associated with walking compared to other static temporal features. However, the algorithm’s accuracy diminishes when cattle are engaged in complex activity states.

#### 3.3.2 Validation of automated step count against manual counting

**FIGURE 8:**
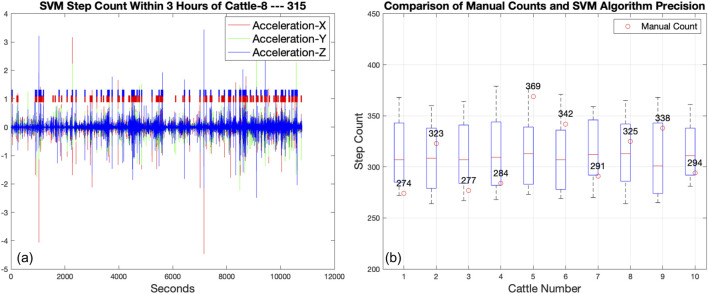
**(A)** The walking behavior of cattle $C_{8}$ , as automatically labeled by SVM for 3 h time window. Continuous walking states are denoted with different colored markers, with the total count is 315 steps. **(B)** A comparison between manually counted steps and 10 automated SVM step counts for all the 10 cattles, all with a statistical time window of 3 h.

Figure 8A illustrates the activity pattern of a specific cow, designated as $C_{8}$ , over a 3 h period, as identified by a Support Vector Machine (SVM) algorithm. The SVM classifier has been trained to recognize continuous walking behavior, which is indicated by the accumulation of markers in the graph. Each marker color represents a different axis of acceleration—X, Y, and Z—captured by a tri-axial accelerometer. The SVM algorithm identified a total of 315 steps during this period. The graph exhibits the characteristic high-frequency noise associated with raw accelerometer data, but the SVM’s algorithmic filtering has successfully distilled this into a clear step count. This pattern of activity, captured in discrete time intervals, provides valuable insights into the locomotion and wellbeing of the animal.

In Figure 8B, offers a side-by-side comparison of SVM algorithm and manual step counts for 10 cattle, labeled $C_{1}$ through $C_{10}$ . Median SVM step counts, denoted by the horizontal line in each boxplot, generally align with manual counts but display variances for $C_{4}$ and $C_{10}$ , with discrepancies of −13 and +16 steps respectively. The interquartile range (IQR) indicates variability in the SVM’s performance, with $C_{4}$ ’s IQR spanning from 270 to 310 steps, suggesting a tendency of the SVM to undercount. Outliers, such as those observed for $C_{2}$ and $C_{9}$ , hint at occasional bursts of activity not consistently captured by the SVM. For example, $C_{2}$ ’s manual count exceeds the SVM’s upper quartile by about 40 steps, indicating possible underdetection of rapid movements by the algorithm. The analysis reveals a mean absolute deviation of around nine steps across the dataset, highlighting the need for refinement in the SVM’s calibration to ensure it captures the full range of cattle movement with accuracy comparable to manual counting.

#### 3.3.3 Comparative performance of classification algorithms

**FIGURE 9:**
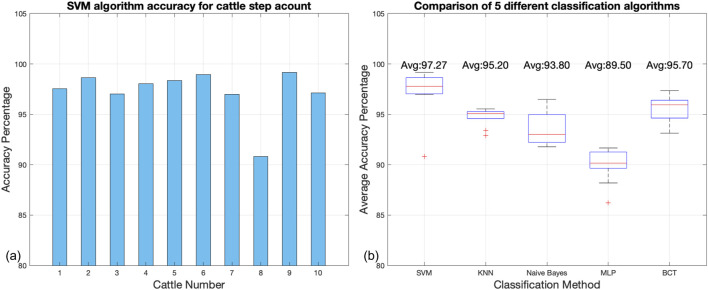
**(A)** Results of step counting experiments conducted using the SVM algorithm for 10 cattle. **(B)** Comparison of the classification performance of five different supervised learning classification algorithms on data collected from wearable devices designed in this paper.

As depicted in Figure 9A, the SVM algorithm’s accuracy for counting cattle steps is evaluated across a sample of 10 cattle. The bar chart shows that the accuracy remains consistently high for most cattle, exceeding 95%. Notably, cattle numbers 1 through 7 and nine show accuracies ranging from 95.5% to 98%, with cattle number 5 reaching the highest at 98%. However, there is a marked decrease for cattle number 8, where the accuracy drops to approximately 87%. This outlier suggests potential discrepancies in step pattern recognition or individual differences in cattle behavior that could affect the SVM’s performance.

Figure 9B presents a comparative analysis of five different classification algorithms used to process data from wearable devices on cattle. The boxplot illustrates the average accuracy percentage achieved by each algorithm, with SVM showing superior performance with an average accuracy of 97.27%. K-Nearest Neighbors (KNN) and Boosted Classification Trees (BCT) also perform well, with average accuracies of 95.20% and 95.70%, respectively. Naive Bayes and Multi-Layer Perceptron (MLP) algorithms exhibit lower average accuracies of 93.80% and 89.50%, respectively, with MLP displaying the widest interquartile range, indicating higher variability in its performance.

The outliers in the SVM and Naive Bayes methods are worth noting, as they may indicate instances where the algorithms significantly deviated from their average performance. The high average accuracy and relatively tight interquartile range of the SVM algorithm underscore its robustness and reliability for this application.

This analysis provides a quantitative assessment of the SVM algorithm’s accuracy for step counting in cattle and compares it with other classification methods. It is evident from the data that while the SVM algorithm performs with high accuracy for most cattle, individual variations do exist, and the algorithm outperforms other classification methods on average.

## 4 Conclusion and discussion

In this investigation, we have successfully demonstrated the functionality of a novel wearable health monitoring device tailored for cattle, marking a substantial contribution to precision livestock farming. Our integration of energy, core, and storage modules within an ergonomically designed, curved shell has proven effective for non-invasive monitoring of cattle body temperature, a critical indicator for early detection of potential health issues. Our experiments on a cohort of 10 cattle have not only validated the effectiveness of the integrated sensors in recording accurate temperature data but have also provided a rich dataset for the analysis of cattle behavior through motion data. The robust 1 s rolling median filtering method applied to the IRT data successfully minimizes environmental noise, ensuring reliable temperature readings. The machine learning algorithms, especially the SVM, have demonstrated high accuracy in classifying various cattle behaviors and counting steps, which are vital indicators of animal wellbeing.

The SVM algorithm’s superior performance in step counting, with an average accuracy of 97.27%, reflects its robustness and adaptability. Although variances were noted in individual cases, such as cattle number 8, these are attributed to behavioral idiosyncrasies and highlight the need for further algorithmic refinement. Comparatively, the SVM outperformed other classification methods, including KNN, Naive Bayes, MLP, and BCT, in both accuracy and consistency. The results underscore the critical role of quality data in training machine learning models and the potential of these technologies in transforming livestock management practices.

Despite these achievements, the study revealed the device’s susceptibility to sunlight, indicating a need for design improvements. Future modifications may include resizing the sensor and adapting its shape to better suit the cattle’s neck contours, enhancing data accuracy and device wearability. To further augment the device’s capabilities, subsequent research should focus on refining the artificial intelligence algorithms for more precise temperature data interpretation. Anticipating temperature-related health abnormalities early can offer farmers a proactive approach to animal healthcare, potentially averting economic losses due to disease.

The research paves the way for future innovations, including the implementation of an embedded behavior classifier within the device’s system, utilizing the most effective machine learning models identified in this study. Expansion to include a variety of sensor types is also anticipated, aiming to develop a versatile and economical solution adaptable to diverse dairy farming practices. In sum, while the current device stands as a significant advancement, it is the springboard for future research that will aim to perfect its design and functionality. The continued evolution of this technology is expected to solidify its place as an essential tool for modern agriculture, ensuring the health and wellbeing of livestock on a large scale.

## Data Availability

The raw data supporting the conclusions of this article will be made available by the authors, without undue reservation.
